# Comparative neuroprotective and exercise capacity effects of prophylactic intermittent fasting and probiotics in sleep-deprived rats: insights into anti-inflammatory marker modulation and *CLOCK* gene regulation

**DOI:** 10.3389/fphar.2026.1796727

**Published:** 2026-06-10

**Authors:** Eman I. Elgizawy, Reda A. A. Abo-Elsoud, Anwar M. Shaban, Hala F. M. Kamel, Hiba S. Al-Amodi, Shimaa Abdelsattar, Rana T. Hikal, Shaimaa M. Hassan

**Affiliations:** 1 Medical Physiology Department, Faculty of Medicine, Menoufia University, Shebin El Kom, Egypt; 2 Medical Physiology Department, Faculty of Medicine, Menoufia University, Menoufia National University, Menoufia, Egypt; 3 Medical Biochemistry and Molecular Biology Department, Faculty of Medicine, Ain Shams University, Cairo, Egypt; 4 Biochemistry Department, Faculty of Medicine, Umm Al-Qura University, Makkah, Saudi Arabia; 5 Clinical Biochemistry and Molecular Diagnostics Department, National Liver Institute, Menoufia University, Shebin El Kom, Menoufia, Egypt; 6 Histology and Cell Biology Department, Faculty of Medicine, Menoufia University, Shebin El Kom, Egypt

**Keywords:** sleep deprivation, intermittent fasting, probiotics, CLOCK gene, neuroinflammation, hippocampus, glial fibrillary acidic protein, cleaved caspase-3

## Abstract

**Background:**

Prophylactic probiotics and intermittent fasting (IF) substantially modulate the neuropsychological functions and exercise capacity in rats subjected to sleep deprivation (SD). A comparative study was conducted to analyze the effects of probiotics and IF on SD-induced neuropsychological disturbances and compromised muscle endurance.

**Methods:**

Forty albino Wistar rats were randomly assigned to four groups. The NSD group was maintained on a standard chow diet for 12 weeks. The SD group followed an SD regimen for 72 h per week over 8 weeks, starting from the fifth week. The SDP group received probiotics at a dose of colony-forming units (CFUs)/100 g/day for 4 weeks prior to SD, followed by 8 weeks of concurrent probiotic administration with SD. The SDIF group underwent an alternate-day fasting regimen for 4 weeks before SD, followed by 8 weeks of simultaneous SD combined with IF. Neuropsychological functions and exercise capacity were tested, and then the brains were carefully dissected, sectioned, and processed for hematoxylin and eosin, cresyl violet, and immunohistochemical staining.

**Results:**

Inflammatory markers, including interleukin-6 (IL-6), tumor necrosis factor-α (TNF-α), and hippocampal expression of the circadian locomotor output cycles kaput (*CLOCK*) gene, were significantly elevated in the SD group. Conversely, it showed significant decreases in endurance, exploratory behavior, hippocampal superoxide dismutase (SOD) activity, and fecal short-chain fatty acids (SCFAs). Histological analysis also revealed hippocampal gliosis, apoptosis, CA1 pyramidal cell degeneration, layer disorganization, and upregulation of glial fibrillary acidic protein (GFAP), NF-κB, and cleaved caspase-3. Nevertheless, both probiotics and IF markedly reduced serum MDA, hippocampal *CLOCK* gene expression, gliosis, and apoptosis and enhanced memory performance. In addition, they significantly increased hippocampal SOD activity and SCFAs.

**Conclusion:**

These findings indicate that prophylactic probiotics decrease cognitive disruption and impaired muscle endurance caused by SD through *CLOCK* gene regulation compared to that with IF. This highlights the need for further research to elucidate these mechanisms. Histological findings also supported these results, showing improved neuronal structure in the hippocampus following probiotic treatment.

## Graphical Abstract

**Figure:**
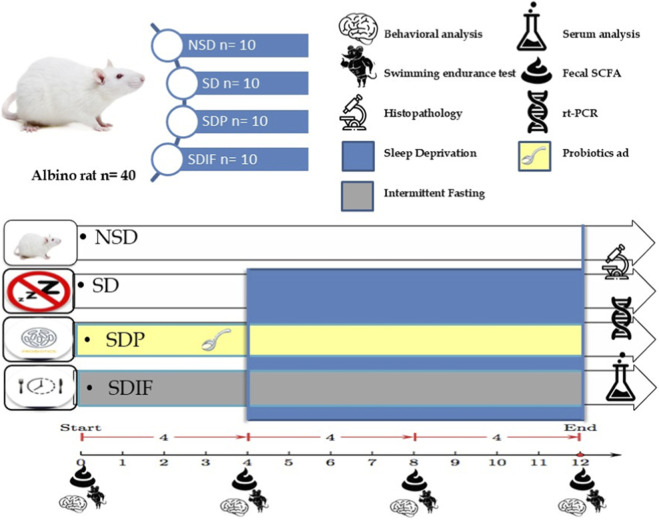
Rats were randomly placed in the NSD, SD, SDP, and SDIF groups. During the experiment, this study evaluated the exercise performance and behavior analyses; measured fecal SCFAs, serum analyses, and hippocampal gene expression; and conducted histopathology and immunohistochemical analyses. NSD, non-sleep-deprivation group; SD, sleep deprivation; SDP, sleep-deprived on probiotics regimen group; SDIF, sleep-deprived on intermittent fasting regimen group.

## 1 Introduction

Sleep deprivation (SD) has been linked to a poor quality of life (Chung et al., 2023), and there is an approximately 14% higher risk of mortality for individuals sleeping less than 7 h per night (Ungvari et al., 2025). SD is a severe stressor, and its allostatic load can lead to cognitive impairments (McEwen, 2006) and reduced muscular endurance (Rault et al., 2024). Chronic SD is connected to cardiovascular complications (Evbayekha et al., 2022), cognitive decline (Han et al., 2024), metabolic imbalances (Jain et al., 2012), and a compromised immune response (Garbarino et al., 2021). These impairments lead to unhealthy aging and age-related pathologies such as diabetes, dementia, and cancer (Carroll and Prather, 2021). It enhances oxidative stress, increases inflammation, and causes hormonal imbalances (Ungvari et al., 2025). In addition to disrupting natural circadian cycles, SD alters the expression of core clock genes, which are crucial for maintaining immunological homeostasis (Zeng et al., 2024). SD disrupts the rhythmic expression of the central and peripheral clock genes, including *CLOCK* and *BMAL1*, causing dysregulation of inflammatory cytokines such as tumor necrosis factor-α (TNF-α) and interleukin-6 (IL-6) (Rodrigues et al., 2023; Huang et al., 2025).

Probiotics are “live microorganisms that, when administered in adequate amounts, confer a health benefit on the host (Sanders, 2008). These are types of bacteria, such as *Lactobacillus*, *Bifidobacterium*, and *Saccharomyces*, that are found in dietary supplements, fermented foods, or specialized products. It may improve the body’s health by modulating gut microbiota, regulating epithelial gut barrier integrity, and enhancing immune responses (Amin et al., 2025). It may also improve lactose digestion and manage inflammatory bowel disease, metabolic syndrome, and even neuropsychiatric conditions via the gut–brain axis (GBA). Given the established potential of gut microbiota modulation to influence neurological outcomes, an established blend of *Lactobacillus* strains (*L. fermentum* and *L. delbrueckii*) at a dosage validated by Liu et al. (2019) was implemented 4 weeks before the SD to ensure sufficient microbiota modulation, allowing us to evaluate the preventative potential of these probiotics against SD-induced deficits via the GBA.

Probiotics’ mechanisms of action could modulate cytokine profiles and short-chain fatty acid (SCFA) production (Plaza-Diaz et al., 2019). Probiotics are also recognized for their systemic and neurocognitive effects (Lin F. L. et al., 2023). Probiotics can alleviate sleep disturbance-related mood symptoms (Liu et al., 2025) by enhancing SCFA production, modulating neurotransmitter pathways (serotonin and GABA), and strengthening the intestinal and blood–brain barriers (Li et al., 2024). Probiotics have been administered to improve sleep quality (Irwin et al., 2020).

On the other hand, intermittent fasting (IF), a diet plan that includes alternating periods of eating and abstaining from food, involves popular regimens such as alternate-day fasting, the 5:2 plan, and time-restricted feeding (TRF) (Patterson and Sears, 2017). IF supports the loss of weight, improves insulin sensitivity, enhances metabolic health, and reduces the risk of heart disease (Vasim et al., 2022). Furthermore, IF affects circadian rhythms (Daas and de Roos, 2021), oxidative stress, and inflammatory processes (Reddy et al., 2024). Moreover, IF affects the gut microbiome (Liu et al., 2021), helping beneficial bacteria grow and increasing the production of SCFAs, which enhance the immunological and neurocognitive functions (Hein et al., 2025). These findings indicate that IF improves nutrition and is a potential treatment that can affect the body.

Thus, in the current study, we aimed to compare the prophylactic effects of probiotics and IF on neuropsychological functions and exercise capacity, their relationship to the CLOCK gene in experimentally induced SD in rats, and the possible molecular mechanisms mediating their action by using various physiological, histological and immunohistochemical methods.

## 2 Materials and methods

The experimental protocol obtained endorsement under approval number 6/2023PHYS16 from the local ethics committee at the Faculty of Medicine, Menoufia University. This was conducted based on the guidelines delineated in the Guide for the Care and Use of Laboratory Animals (National Academies Press, eighth edition) (Council et al., 2010).

### 3.1 Experimental design

Forty adult male Wistar albino rats weighing between 100 and 150 g and aged between 5 and 6 weeks were used in this study. Throughout the study, all rats had unrestricted access to food and water after a 2-week conditioning period under stable environmental conditions, temperature of 22 °C ± 2 °C, and humidity of 40%–70% with a 12:12-h light/dark cycle (lights on at 07:00). The rats were divided into four experimental groups (10/group):

Non-sleep deprivation group (NSD): In this group, rats were administered a conventional rat chow diet over a duration of 12 weeks (Hazzaa et al., 2020).

Sleep deprivation group (SD): In this group, rats were sleep deprived for a total of 72 h SD per week for 8 weeks, starting from the fifth week, by the modified multiple platform method (MMPM) (Chung et al., 2023).

Sleep-deprived on probiotics regimen group (SDP): In this group, before being subjected to SD, rats were administered probiotic supplements for four consecutive weeks at a dosage of one billion colony-forming units (CFUs)/100 g/day of *Lactobacillus fermentum* and *Lactobacillus delbrueckii* by oral gavage; this choice and regimen are strategically based on the robust protocols established in prior literature (Liu et al., 2019; Azagra-Boronat et al., 2020) to ensure the symbiotic benefits of these species. Then, 8-week chronic SD (total of 72 h SD per week), as in the SD group, was induced continually and simultaneously with probiotics for the subsequent weeks.

**FIGURE 1:**
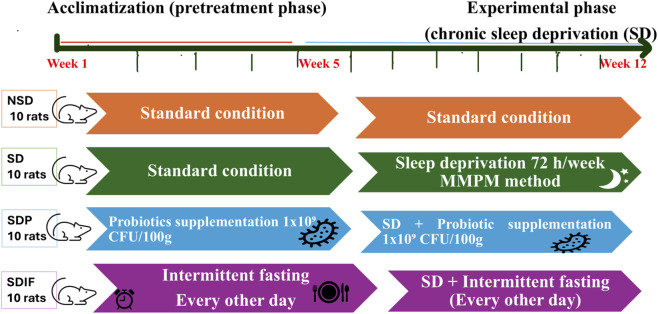
Experimental timeline illustrating a 4-week pretreatment phase followed by 8 weeks of chronic sleep deprivation. Rats were assigned to four groups, namely, normal sleep (NSD), sleep deprivation (SD), sleep deprivation with probiotic treatment (SDP), and sleep deprivation with intermittent fasting (SDIF).

Sleep-deprived on intermittent fasting regimen group (SDIF): In this group, before being subjected to SD, rats followed a dietary regimen IF) in which food was available only every other day for four consecutive weeks (Hazzaa et al., 2020). Then, 8-week chronic SD (total 72 h SD per week), as in the SD group, was induced continually and simultaneously with IF for the subsequent weeks (Figure 1).

### 3.2 Sampling

Behavioral assessments (using open-field test, Y-maze, and tail suspension test) and endurance evaluations were conducted, with fecal specimens being acquired for SCFA analysis at the initiation of the study, the conclusion of the fourth and eighth weeks, and after 12 weeks (marking the experiment’s conclusion) from the onset of the study. The subjects underwent an overnight fasting period, subsequent to which the samples of blood were procured from the retro-orbital venous plexus. Serum samples were subsequently isolated and utilized for the quantification of serum pro-inflammatory markers, specifically IL-6, (TNF-α), and malondialdehyde (MDA). The rats were euthanized via cervical decapitation under anesthesia (achieved through an intraperitoneal administration of 60 mg/kg phenobarbital), and their brains were classified into two segments. One segment was placed at −80 °C for the relative quantification of circadian locomotor output cycles kaput *CLOCK* gene expression within hippocampal tissues and for the evaluation of superoxide dismutase (SOD) activity. The other segment was placed in 10% neutral formalin for conducting histopathological and immunohistochemical analyses.

### 3.3 Assessment of behavioral responses

Evaluation was conducted between the hours of 9:00 a.m. and 2:00 p.m. within a quiet room with natural daylight. Before conducting evaluations, the rats were submitted to the observation environment for 1 hour. Each test was controlled using a video recording device, which was a Samsung ST93 Digital Camera manufactured in Suwon, South Korea. The apparatus was sanitized using 70% ethanol to alleviate any olfactory cues that may affect the subjects before each evaluation (Matheus et al., 2016).

### 3.4 Assessment of motor function

#### 3.4.1 Swimming endurance test

The rats were put in a swimming apparatus (100 cm in diameter × 40 cm in depth) full of water maintained at a temperature of 25 °C ± 2 °C to a depth of 30 cm, with weights (5% of body mass) affixed to the tail base of each rat (Xu et al., 2018). The duration of exhaustive swimming was recorded based on the observation of uncoordinated movements in an individual rat, wherein the rat failed to resurface within a timeframe of 5 s.

#### 3.4.2 Open-field test

A wooden arena (100 cm × 100 cm × 60 cm in height, featuring light brown walls and flooring) was classified into 25 squares. The rat was placed in the open arena and permitted to navigate freely for a span of 10 min (Wang et al., 2023a). The number of squares traversed, frequency of grooming behaviors, instances of rearing, total entries into the central zone, and occurrences of freezing behavior were quantified, along with the duration spent in the inner and outer zones.

### 3.5 Assessment of short-Term spatial working memory

#### 3.5.1 Y-maze

The Y-maze consists of three arms, extending approximately 50 cm in length, 10 cm in width, and 20 cm in height. This apparatus evaluates spatial working memory capabilities (Soares et al., 2013). A single rodent was placed near the maze’s center and permitted to explore the Y-maze for a period of 8 min. To facilitate spatial orientation, various distal visual cues (e.g., geometric shapes) were positioned on the room walls around the Y-maze and retained in fixed locations throughout the testing period. Spatial cognition was assessed by calculating the percentage of spontaneous alternation, which is calculated as (the number of successful alternations/(the total number of entries – 2) x 100.

### 3.6 Assessment of depressive-like behavior

#### 3.6.1 Tail suspension test

The tail suspension test (TST) is a predictive measure for depressive-like behavior. Rats were hung 1 cm from the tip by their tails for a short duration (6 min) at a height of 25 cm above the ground (Can et al., 2012; Pignataro et al., 2023). The period of immobility was recorded, which is defined as the state where the rat exhibited passivity without any observable movement. Adult male Wistar albino rats weighing between 100 and 150 g and aged between 5–6 weeks were used in this study. This specification was chosen to ensure the safety and suitability of the test.

#### 3.6.2 Biochemical analysis

The serum IL-6 value was measured using ELISA commercial kits (DI develop, Canada) according to Brianza-Padilla et al. (2018).

The serum TNF-α was estimated using ELISA kits (Biotech, Shanghai, China) according to Li et al. (2020).

The serum MDA concentration was quantified using spectrophotometric methods combined with commercially available kits (Biodiagnostic Company, Cairo, Egypt) according to the protocol delineated by Draper et al. (1993).

The fecal SCFA was measured by gas chromatography–mass spectrometry (GC–MS) after derivatization to the corresponding pentafluorobenzyl bromide (PFBBr) derivative, as detailed by Hussein et al. (2014).

#### 3.6.3 Measurement of hippocampal tissues SOD

Hippocampal tissues were homogenized in a normal saline solution at a ratio of 1:9 (w/v). The resultant homogenate was centrifuged at 1,800 *g*/min for 10 min. The supernatant was used to quantify SOD. The concentration of SOD was evaluated spectrophotometrically using commercial kits (Biokit Company, Egypt) according to the illustrated methodology in Liu et al. (2007).

### 3.7 Quantitative assay of *CLOCK* gene expression using the reverse transcriptase polymerase chain reaction technique (RT-PCR)

Hippocampal tissues were used for the isolation of RNA using the PureLink^TM^ RNA Mini Kit (Life Technologies). The quality and purity of the RNA were confirmed. The RNA was stored at −80 °C until further use. The first stage involved the synthesis of complementary DNA (cDNA) using the G-Storm Thermal Cycler (United Kingdom) combined with a High-Capacity cDNA Reverse Transcription Kit (Applied Biosystems, Foster City, CA, United States) for a single cycle. Primers specific to GAPDH were used as an RNA loading control during RT-PCR reactions. The second stage included the amplification of cDNA; cDNA was utilized in SYBR green-based quantitative real-time PCR for the relative quantification (RQ) of circadian locomotor output cycles kaput (*CLOCK*) gene expression, facilitated by the SensiFAST^TM^ SYBR Lo-ROX Kit (United States), utilizing the following specifically designed primers (Biokit, Egypt): the forward primer for *CLOCK* was (5-TCA​CCA​CGT​TCA​CTC​AGG​ACA-3) and the reverse primer was (5-AAGGATTCCCA TGGAGC AA-3). The analysis was performed using Applied Biosystems 7500 software version 2.0.1. The RQ of the *CLOCK* gene expression was defined using the comparative ΔΔCt method, where the *CLOCK* gene mRNA was normalized to an endogenous reference gene (*GAPDH*) compared to a control, which is pursuant to the guidelines established in Koritala et al. (2021).

### 3.8 Histological studies

Brain tissues were removed, and parasagittal sections were prepared. The samples were fixed in a 4% solution of formaldehyde for 24 h after dehydration through a graded series of increasing alcohol concentrations. The tissues were subjected to a clearing process using xylene and were embedded in paraffin blocks. The paraffin-embedded brain blocks were sectioned into to a thickness of 4 μm, deparaffinized, and affixed to glass slides for the following procedures:

1. Hematoxylin and eosin (H&E) staining was performed to illustrate the history of architecture of the hippocampus, as described by Shalaby et al. (2024).
2. Cresyl violet staining was performed to determine viable surviving neurons that were defined by spherical open-faced nuclei, as indicated by Suvarna et al. (2018).
3. Immunohistochemical staining was conducted using antibodies against glial fibrillary acidic protein (GFAP) as an astrocytic marker for the assessment of gliosis; cleaved caspase-3 as an indicator of apoptosis; and nuclear factor-kappa β (NF-kβ), IL-6, and TNF-α as markers of inflammation, as described by Fareed et al. (2022) and Shalaby et al. (2023).

The slides were incubated in a 3% solution of hydrogen peroxide in phosphate-buffered saline (PBS) for 30 min to mitigate endogenous peroxidase activity. Antigen retrieval was performed by heating the slides in sodium citrate (10 mM, pH 6.0) at 95 °C for 15 min. They were incubated for 1 h at room temperature in PBS supplemented with 10% normal serum to block non-specific binding.

The slides were incubated overnight at 4 °C with the following antibodies: cleaved caspase-3 (rabbit polyclonal, 1:300, #9662, Cell Signaling Technology, Danvers, MA, United States), GFAP (mouse monoclonal, 1:200, SC-58766, Santa Cruz Biotechnology, Inc.), IL-6 (rabbit polyclonal, 1:1,200, Wako, Osaka, Japan), TNF-α (rabbit polyclonal, Catalog No. A11534, ABclonal Technology, 1:100), and NF-κB p65 (polyclonal, Catalog No. bs-20159R, Bios Antibodies).

The relevant antibodies were administered to the tissue sections after applying the streptavidin–biotin detection methodology for 20 min. The slides underwent treatment with diaminobenzidine (DAB) and were counterstained utilizing Mayer’s hematoxylin. Each section was scrutinized under a light microscope, and images were systematically captured.

The GFAP-positive cells were identified by brown staining of the cell membrane and cytoplasm responding to astrocytes.

NF-κB-positive cells, primarily pyramidal neurons, were recognized by brown staining of both the cytoplasm and nucleus of hippocampal pyramidal neurons.

TNF-α-positive cells, which are also pyramidal neurons, were identified by brown staining of the cytoplasm of hippocampal pyramidal neurons.

IL-6-positive cells were revealed by brown cytoplasmic staining in hippocampal pyramidal neurons.

Cleaved caspase-3-positive cells, which are indicative of apoptosis, were identified by brown staining of their nuclei.

### 3.8 Morphometric study

All slides from each animal were analyzed using a conventional light microscope equipped with a ×40 objective. For each animal, three representative slides were prepared, and five non-overlapping fields were photographed using a Nikon E400 digital microphotography system (N150, Nikon, Tokyo, Japan). A total of 10 animals per group were included in the analysis across the four experimental groups. The captured images were analyzed using Digimizer software, version 4.6.1 (MedCalc Software Ltd., Acacialaan, Belgium) (Wang et al., 2005). The images were analyzed to evaluate the following:

- The mean number of degenerated pyramidal cells within the CA1 region of the hippocampus (H&E, ×200).
- The mean number of viable pyramidal cells in the CA1 region of the hippocampus (cresyl violet, ×200).
- The mean area percentage of GFAP expression (×400).
- The mean area percentage of NF-κB expression (×400).
- The mean area percentage of TNF-α expression (×400).
- The mean area percentage of IL-6 expression (×400).
- The mean number of cleaved caspase-3-positive cells (×400).

### 3.9 Statistical analysis

Data were shown as the mean ± SD. SPSS version 26 for Windows (SPSS Inc., Chicago, Illinois, United States) was used for statistical analysis. ANOVA and *post-hoc* multiple comparison tests were performed to determine the significance of group differences, and statistical significance was defined in all experimental trials when the *p*-value was ≤ 0.05. The SD values presented represent the variability among individual animals within the same experimental group for this single cohort. No additional independent experimental repetitions were performed; therefore, the reported variability reflects only within-cohort variation.

## 4 Results

### 4.1 Swimming endurance test

**FIGURE 2:**
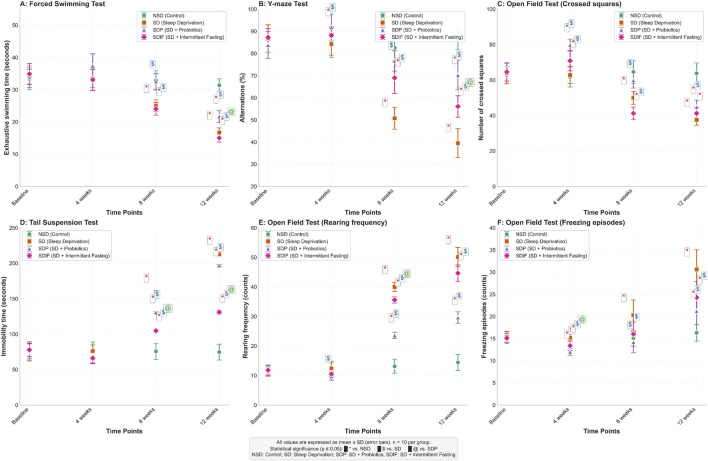
**(A)** Effects of probiotics and IF on exhaustive swimming time. **(B)** Effects of probiotics and IF on the percentage of alterations in the Y-maze (% alternation). **(C)**. Number of crossed squares. **(D)**. Effects of probiotics and IF on immobility time (sec) in the tail suspension test (immobility time). **(E)**. Rearing frequency and **(F)**. freezing episodes in OFT after 4, 8, and 12 weeks from the beginning of the study in experimentally induced SD. NSD, control group; SD, sleep deprivation; SDP, sleep deprivation with probiotics; SDIF, sleep deprivation with intermittent fasting. Error bars represent SD to indicate variability within each group.

The results of our study showed that SD significantly decreased the exhaustive swimming time after 8 weeks (Figure 2A, F (3, 36) = 41.150; *p* < 0.001) and 12 weeks (Figure 2A, F (3, 36) = 191.507; *p* < 0.001) from the beginning of the study relative to NSD. Moreover, probiotics significantly increased (*p* < 0.001) the exhaustive swimming time after 8 and 12 weeks from the beginning of the study relative to the SD and SDIF. IF insignificantly (*p* > 0.05) changed the exhaustive swimming time relative to SD rats throughout the experiment (Figure 2A; Table 1).

**TABLE 1:** Results of the forced swimming tests (exhaustive swimming time) and Y-maze test at different intervals among studied groups (n = 40).

Parameter	Non-sleep-deprivation group (n = 10)	Sleep deprivation group (n = 10)	Sleep-deprived on probiotics regimen group (n = 10)	Sleep-deprived on intermittent fasting regimen group (n = 10)
Exhaustive swimming time at baseline (sec)	33.40 ± 3.41	33.95 ± 2.36	34.06 ± 3.34	34.92 ± 3.25
F = 0.409, *p*-value = 0.748				
Exhaustive swimming time after 4 weeks (sec)	33.49 ± 2.74	33.42 ± 3.57	37.44 ± 3.69	33.07 ± 3.36@
F = 0.3.778, *p*-value =0.019*				
Exhaustive swimming time after 8 weeks (sec)	32.86 ± 3.02	25.02 ± 1.83*	32.58 ± 2.39$	24.02 ± 1.94*@
F = 41.150, *p*-value <0.001*				
Exhaustive swimming time after 12 weeks(sec)	31.38 ± 1.97	16.68 ± 1.46*	21.71 ± 1.91*$	15.02 ± 1.29*@
F = 191.507, *p*-value <0.001*				
Behavioral Y-maze test				
% of alternations at baseline	87.17 ± 4.11	86.73 ± 6.19	83.76 ± 6.11	87.19 ± 4.03
F = 0.999, *p*-value = 0.404				
% of alternations after 4 weeks	84.84 ± 6.66	84.16 ± 5.00	91.77 ± 6.03*$	88.14 ± 3.87
F = 4.043, *p*-value =0.014*				
% of alternations after 8 weeks	82.51 ± 5.79	50.75 ± 4.93*	76.61 ± 4.57$	69.00 ± 7.01*$
F = 59.574, *p*-value <0.001*				
% of alternation after 12 weeks	86.34 ± 4.70	39.48 ± 6.54*	70.22 ± 6.60*$	56.04 ± 4.85*$@
F = 121.075, *p*-value <0.001*				

Data were expressed as the mean ± standard deviation (SD); F: one-way ANOVA test, and statistical significance was taken as *p* ≤ 0.05 for all experiments. *: significance compared to the corresponding value of the non-sleep deprivation group, $: significance compared to the corresponding value of the sleep deprivation group, and @: significance compared to the corresponding value of the sleep-deprived on probiotics regimen group.

### 4.2 Y-maze test

The results of the Y-maze test showed that SD significantly decreased the percentage of alternations after 8 weeks (Figure 2B, F (3, 36) = 59.574; *p* < 0.001) and 12 weeks (Figure 2B, F (3, 36) = 121.075; *p* < 0.001) from the start of the study according to NSD rats. Moreover, probiotics increased (*p* < 0.05) the percentage of alternations after 4 weeks (Figure 2B, F (3, 36) = 4.043; *p* < 0.05), 8 weeks, and 12 weeks from the beginning of the study relative to SD rats and significantly increased (*p* < 0.05) the percentage of alternations after 8 and 12 weeks from the beginning of the study relative to SDIF. IF significantly (*p* < 0.001) increased the percentage of alterations after 8 and 12 weeks relative to the SD rats (Figure 2B; Table 1).

### 4.3 Open-field test

SD significantly increased rearing frequency, frequency of grooming, and freezing episodes after 8 weeks (F (3, 36) = 579.857; *p* < 0.001; F (3, 36) = 4.502; *p* < 0.05; F (3, 36) = 10.923; *p* < 0.001) and 12 weeks (F (3, 36) = 346.110; *p* < 0.001; F (3, 36) = 3.045; *p* < 0.05; F (3, 36) = 31.614; *p* < 0.001) from the start of the study compared to that in NSD rats. In contrast, it decreased the number of crossed squares and time in the center after 8 weeks (F (3, 36) = 50.538; *p* < 0.001; F (3, 36) = 15.401; *p* < 0.001) and decreased the number of crossed squares, time, and total entries to the center after 12 weeks (F (3, 36) = 77.360; *p* < 0.001; F (3, 36) = 82.364; *p* < 0.001; F (3, 36) = 17.827; *p* < 0.001) from the beginning of the study compared to that in NSD rats. Probiotics decreased (*p* < 0.05) rearing frequency, frequency of grooming, and freezing episodes after 8 and 12 weeks from the beginning of the study compared to that in the SD rats. In contrast, it significantly (*p* < 0.05) increased the number of crossed squares and time in the center after 4, 8, and 12 weeks from the beginning of the study compared to that in SD rats. However, IF significantly decreased (*p* < 0.05) rearing frequency and freezing episodes after 8 and 12 weeks from the beginning of the study relative to that in the SD rats, while it significantly (*p* < 0.05) increased the number of crossed squares after 4 and 8 weeks from the beginning of the study relative to that in the SD rats. Moreover, IF significantly increased (*p* < 0.05) rearing frequency after 8 and 12 weeks from the beginning of the study relative to the SDP rats. It significantly (*p* < 0.05) reduced the number of crossed squares after 4 and 8 weeks from the beginning of the study compared to that in SDP rats (Figures 2C,E,F; Table 2).

**TABLE 2:** Behavioral test (OFT and TST) at different intervals among the studied groups (n = 40).

Parameter	Non-sleep-deprivation group (n = 10)	Sleep deprivation group(n = 10)	Sleep-deprived on probiotics regimen group (n = 10)	Sleep-deprived on intermittent fasting regimen group (n = 10)
Number of crossed squares at baseline	62.9 ± 4.86	63.8 ± 5.88	66.6 ± 2.88	64.6 ± 5.15
F = 1.070, *p*-value =0.374				
Number of crossed squares after 4 weeks	61.7 ± 5.62	62.8 ± 4.80	79.7 ± 3.40*$	70.9 ± 5.70*$@
F = 28.306, *p*-value <0.001*				
Number of crossed squares after 8 weeks	64.6 ± 6.43	50.0 ± 3.71*	59.8 ± 4.29$	41.2 ± 3.52*$
F = 50.538, *p*-value <0.001*				
Number of crossed squares after 12 weeks	63.8 ± 5.88	37.5 ± 3.06*	44.8 ± 3.79*$	41.2 ± 3.52*
F = 77.360, *p*-value <0.001*				
Rearing frequency at baseline	11.6 ± 1.26	11.2 ± 1.03	11.5 ± 1.78	11.8 ± 1.75
F = 0.281, *p*-value =0.839				
Rearing frequency after 4 weeks	12.0 ± 2.26	12.4 ± 2.37	9.80 ± 1.43$	10.5 ± 1.62
F = 3.923, *p*-value =0.016*				
Rearing frequency after 8 weeks	13.1 ± 2.38	40.0 ± 1.49*	23.6 ± 1.07*$	35.6 ± 1.07*$@
F = 579.857, *p*-value <0.001*				
Rearing frequency after 12 weeks	14.4 ± 2.76	50.1 ± 3.25*	29.6 ± 1.96*$	44.6 ± 2.80*$#
F = 346.110, *p*-value <0.001*				
Number of grooming at baseline	25.6 ± 1.51	27.0 ± 3.06	27.0 ± 3.06	28.5 ± 3.57
F = 1.667, *p*-value =0.191				
Number of grooming after 4 weeks	27.3 ± 2.91	25.6 ± 1.65	24.3 ± 2.67	26.9 ± 3.31
F = 2.526, *p*-value =0.73				
Number of grooming after 8 weeks	29.6 ± 5.36	38.4 ± 6.95*	30.6 ± 5.60$	33.3 ± 5.44
F = 4.502, *p*-value =0.009*				
Number of grooming after 12 weeks	30.1 ± 5.74	38.0 ± 7.83	30.4 ± 6.24	34.2 ± 6.96
F = 3.045, p-value =0.054				
Time in the center at baseline	10.2 ± 1.03	9.80 ± 0.63	10.1 ± 0.88	10.0 ± 0.94
F = 0.374, *p*-value =0.772				
Time in the center after 4 weeks	10.1 ± 0.99	10.0 ± 0.94	12.0 ± 1.41*$	12.0 ± 0.94*$
F = 10.650, *p*-value <0.001*				
Time in the center after 8 weeks	8.90 ± 1.52	5.80 ± 0.63*	7.50 ± 1.08*$	6.40 ± 0.97*
F = 15.401, *p*-value <0.001*				
Time in the center after 12 weeks	8.80 ± 1.40	2.90 ± 0.57*	4.10 ± 0.88*$	3.20 ± 0.79*
F = 82.364, *p*-value <0.001*				
Total entries to the center at baseline	6.80 ± 2.57	6.80 ± 2.53	6.70 ± 2.45	6.50 ± 2.42
F = 0.032, *p*-value =0.992				
Total entries to the center after 4 weeks	6.70 ± 2.45	6.70 ± 2.45	7.30 ± 2.16	6.70 ± 2.21
F = 167, *p*-value =0.918				
Total entries to the center after 8 weeks	4.90 ± 2.56	3.80 ± 1.03	4.50 ± 1.35	4.20 ± 1.32
F = 0.775, *p*-value =0.515				
Total entries to the center after 12 weeks	5.60 ± 2.63	1.20 ± 0.92*	1.70 ± 1.06*	81.40 ± 0.97
F = 17.827, *p*-value <0.001*				
Freezing episodes at baseline	15.2 ± 1.32	15.4 ± 1.26	15.0 ± 0.82	15.1 ± 0.99
F = 0.234, *p*-value =0.872				
Freezing episodes after 4 weeks	15.2 ± 0.79	15.2 ± 0.79	12.0 ± 0.8*$2	13.4 ± 1.07*$@
F = 31.435, *p*-value <0.001*				
Freezing episodes after 8 weeks	15.1 ± 1.37	20.3 ± 3.43*	14.2 ± 2.39$	16.0 ± 2.71$
F = 10.923, *p*-value <0.001*				
Freezing episodes after 12 weeks	16.3 ± 1.89	30.6 ± 4.45*	21.2 ± 3.05*$	24.3 ± 3.56*$
F = 31.614, *p*-value <0.001*				
Behavioral test (TST) among studied groups				
Immobility time (sec) at baseline	74.78 ± 11.30	75.93 ± 13.73	76.77 ± 10.73	77.82 ± 9.25
F = 0.128, *p*-value =0.943				
Immobility time (sec) after 4 weeks	76.35 ± 12.61	76.22 ± 8.55	69.10 ± 9.66	66.15 ± 7.86
F = 2.736, *p*-value =0.058				
Immobility time (sec) after 8 weeks	75.57 ± 11.70	159.32 ± 1.86*	130.55 ± 1.13*$	104.77 ± 2.12*$@
F = 350.449, *p*-value <0.001*				
Immobility time (sec) after 12 weeks	74.58 ± 11.20	212.54 ± 2.45*	196.81 ± 1.41*$	130.96 ± 2.65*$@
F = 1146.779, *p*-value <0.001*				

Data were expressed as the mean ± standard deviation (SD); F: one-way ANOVA test, and statistical significance was taken as *p* ≤ 0.05 for all experiments. *: significance compared to the corresponding value of the non-sleep-deprivation group, $: significance compared to the corresponding value of the sleep deprivation group, and @: significance compared to the corresponding value of the sleep-deprived on probiotics regimen group.

### 4.4 Tail suspension test

Regarding TST, SD significantly increased the immobility time (sec) after 8 (Figure 2D, F (3, 36) = 350.449; *p* < 0.001) and 12 weeks (Figure 2D, F (3, 36) = 1146.779; *p* < 0.001) from the beginning of the study compared to that in NSD rats. In contrast, probiotics significantly decreased (*p* < 0.001) the immobility time (sec) after 8 and 12 weeks from the beginning of the study relative to the SD rats. Moreover, IF significantly (*p* < 0.001) decreased immobility time (sec) after 8 and 12 weeks from the beginning of the study compared to that in SD and SDP rats (Figure 2D; Table 2).

### 4.5 Serum MDA and tissue SOD

**FIGURE 3:**
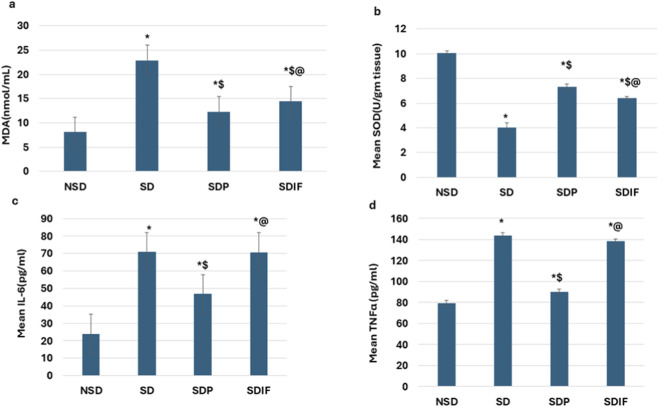
Effects of probiotics and IF on **(a)** serum MDA, **(b)** hippocampal SOD, **(c)** serum IL-6, and **(d)** TNF-α in experimentally induced SD. (*) significant when compared to NSD (*p-*value ≤ 0.05), ($) significant when compared to the SD group (*p-*value ≤ 0.05), and (@) significant when compared to the SDP group (*p* ≤ 0.05), (n = 10).

Concerning oxidative stress markers, SD significantly increased serum MDA (Figure 3a, F (3, 36) = 60.835; *p* < 0.001) but decreased tissue SOD (Figure 3b, F (3, 36) = 1004.054; *p* < 0.001) compared to that in NSD rats. Probiotics and IF decreased (*p* < 0.001) serum MDA, but they increased (*p* < 0.001) tissue SOD compared to that in SD rats. Probiotics significantly increased (*p* < 0.001) tissue SOD and insignificantly changed (*p* > 0.05) serum MDA relative to that in SDIF rats (Figures 3a,b).

### 4.6 Serum IL-6 and TNF-α

Regarding inflammatory markers, SD significantly increased serum IL-6 (Figure 3c, F (3, 36) = 210.797; *p* < 0.001) and TNF-α (Figure 3d, F (3, 36) = 1628.9; *p* < 0.001) relative to that in the NSD group. Probiotics significantly decreased (*p* < 0.001) serum IL-6 and TNF-α compared to that in the SD and SDIF groups. IF insignificantly changed (*p* > 0.05) serum IL-6 and TNF-α according to the SD group (Figures 3c,d).

### 4.7 Fecal short-chain fatty acids assay

**FIGURE 4:**
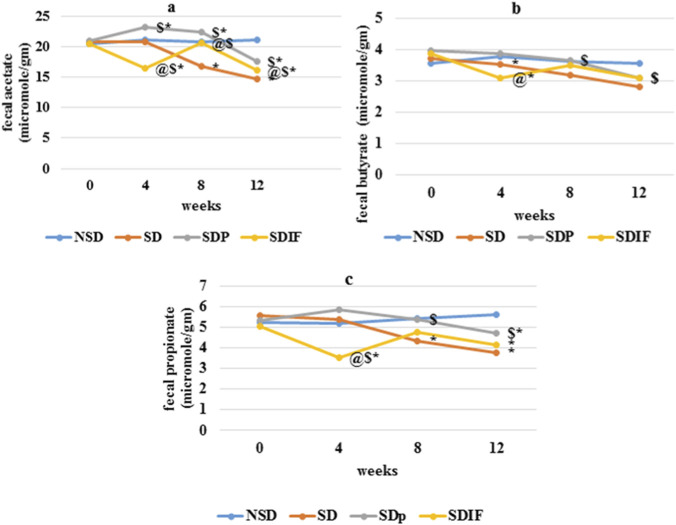
Effects of probiotics and IF on **(a)** fecal acetate, **(b)** fecal butyrate, and **(c)** fecal propionate after 4, 8, and 12 weeks from the beginning of the study in experimentally induced SD (*) significant when compared to NSD (*p-*value ≤ 0.05), ($) significant when compared to the SD group (*p-*value ≤ 0.05), and (@) significant when compared to the SDP group (*p* ≤ 0.05), (n = 10).

SD significantly decreased fecal acetate and propionate after 8 weeks (Figure 4a, F (3, 36) = 24.795; *p* < 0.001; Figure 4c, F (3, 36) = 4.269; *p* < 0.05) and 12 weeks (Figure 4a, F (3, 36) = 104.342; *p* < 0.001; Figure 4c, F (3, 36) = 17.009; *p* < 0.001) from the beginning of the study and decreased fecal butyrate after 12 weeks (Figure 4b, F (3, 36) = 8.446; *p* < 0.001) from the beginning of the study relative to NSD rats. Probiotics significantly increased (*p* < 0.05) fecal acetate after 4, 8, and 12 weeks from the beginning of the study relative to the SD and SDIF rats, while it significantly increased (*p* < 0.05) fecal butyrate and propionate relative to the SD and insignificantly changed (*p* > 0.05) fecal butyrate and propionate relative to SDIF rats after 8 and 12 weeks from the beginning of the study. IF elevated (*p* < 0.05) fecal acetate after 8 and 12 weeks and increased (*p* < 0.05) fecal propionate after 4 weeks from the beginning of the study relative to the SD rats (Figure 4).

### 4.8 Genetic assay (hippocampal *CLOCK* gene expression)

**FIGURE 5:**
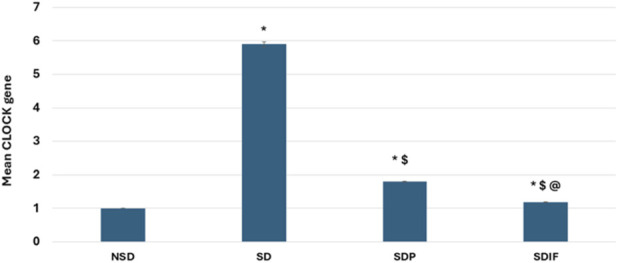
Effect of probiotics and IF on hippocampal *CLOCK* gene in experimentally induced SD (*) significant when compared to NSD (*p-*value ≤ 0.05), ($) significant when compared to the SD group (*p-*value ≤ 0.05), and (@) significant when compared to the SDP group (*p* ≤ 0.05), (n = 10).

SD significantly increased gene expression (Figure 5, F (3, 36) = 4146.779; *p* < 0.001) compared to NSD. Probiotics and IF significantly decreased (*p* < 0.001) gene expression compared to SD. IF decreased (*p* < 0.001) gene expression relative to SDP (Figure 5).

### 4.9 Histopathological findings

**FIGURE 6:**
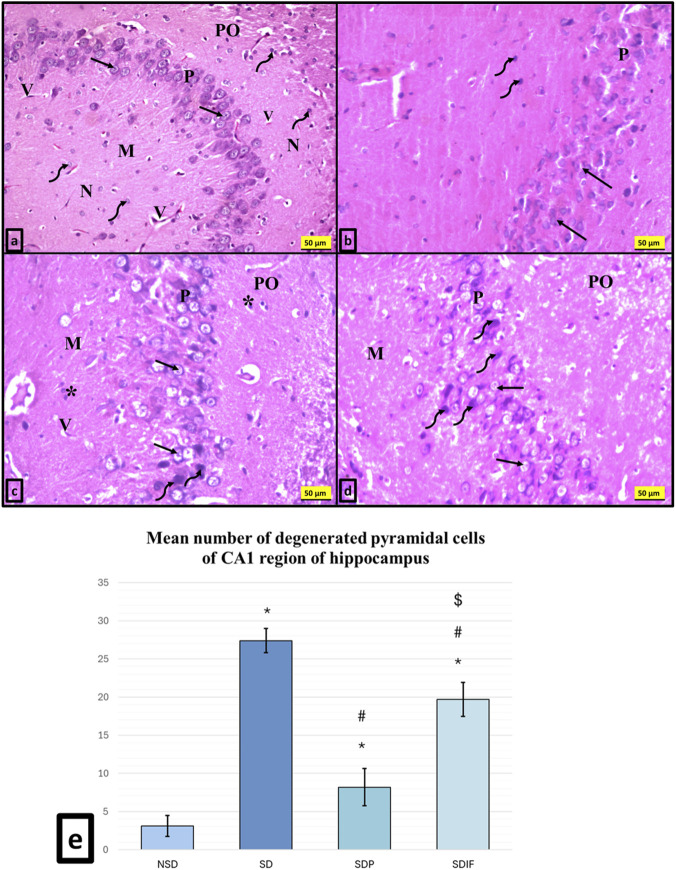
Photomicrographs from the CA1 regions of the hippocampus: **(a)** NSD group shows a normal histological structure of this area of the hippocampus. It is divided into three layers, namely, the inner molecular (M) layers, the middle pyramidal (P) layers, and the outer polymorphic (PO). The pyramidal layer consists of 2–3 rows of closely packed pyramidal cells (arrows). Neuroglial cells (curved arrows) and small blood vessels (V) are distributed throughout the neuropil (N). **(b)** SD group shows the pyramidal layer (P) with multiple degenerated pyramidal cells (arrows). Notice darkly stained neuroglial cells (curved arrows). **(c)** SDP group showing that the CA1 region of the hippocampus is similar to that of the NSD group, displaying its three layers, namely, the outer polymorphic (PO), middle pyramidal (P), and inner molecular (M) layers. Most pyramidal cells appear as large cells with big vesicular nuclei (arrows). The cells of a few pyramidal cells are shrunken, with dark condensed nuclei and dark cytoplasm (curved arrows). Neuroglial cells (asterisks) and blood vessels (v) are observed in the neuropil. **(d)** SDIF group showing the CA1 region of the hippocampus with an apparently normal histoarchitecture, including the three layers, namely, polymorphic (PO), pyramidal (P), and molecular (M). Some pyramidal cells appear normal (arrows), while others are shrunken with darkly stained nuclei (curved arrows). (H&E ×200, scale bar = 50 μm). **(e)** Mean number of degenerated pyramidal cells of the CA1 region of the hippocampus in experimental groups. *Significant versus the NSD group, #significant versus the SD group, and $ significant versus the SDP group, n = 10.

**FIGURE 7:**
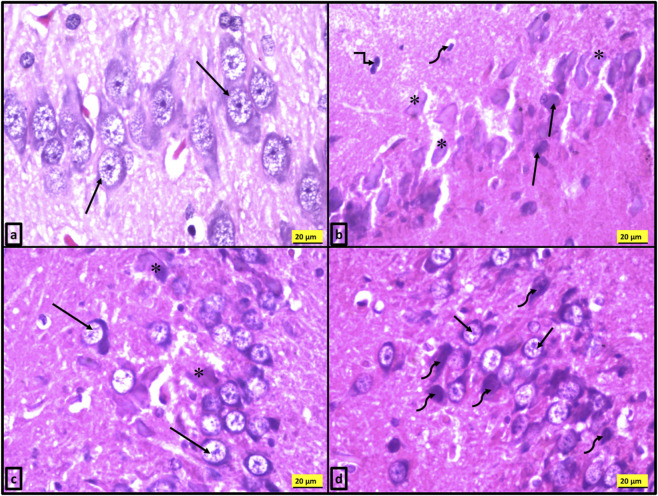
Photomicrographs from the CA1 regions of the hippocampus: **(a)** NSD group shows pyramidal cells (arrows) with vesicular, well-demarcated, rounded nuclei, nucleoli, and scanty basophilic cytoplasm. **(b)** SD group showing that some pyramidal cells are shrunken with darkly stained nuclei (arrows), while others show faintly stained nuclei (asterisks). Perineuronal spaces surround both types. Some nerve cells with stained nuclei are fused (elbow arrow). In addition, some neuroglial cells exhibit darkly stained nuclei (curved arrows). **(c)** SDP group shows that most pyramidal cells are organized in compact rows and possess rounded vesicular nuclei with nucleoli (arrows). A few pyramidal cells are shrunken, with dark condensed nuclei and dark cytoplasm (asterisks). **(d)** SDIF group shows that some pyramidal cells appear normal (arrows), while others are shrunken with darkly stained nuclei (curved arrows). (H&E ×400, scale bar = 20 μm).

H&E-stained sections (Figures 6, 7):

The NSD group demonstrated the normal histological architecture of the Cornu Ammonis 1(CA1) area of the hippocampus. It was divided into three distinct layers, namely, the outer polymorphic, middle pyramidal, and inner molecular layers. The pyramidal layer comprised 2–3 rows of packed pyramidal cells with vesicular, well-demarcated, rounded nuclei, prominent nucleoli, and scanty basophilic cytoplasm. These layers had sparse neuroglial cells with small nuclei and small blood vessels distributed among the neuropil (Figures 6a, 7a).

The area of CA1 in the SD group revealed marked structural alterations as the pyramidal layer consisted of degenerated pyramidal cells. Pyramidal neurons were shrunken with darkly stained nuclei, whereas others exhibited faint, karyolitic nuclei. Both types were surrounded by widened perineuronal spaces. They were displaced, showing deeply stained nuclei that appeared fused. Several neuroglial cells also have darkly stained nuclei (Figures 6b, 7b).

All the histological alterations that occurred in the SD group were ameliorated in the SDP group. The area of CA1 of the hippocampus was similar to that of the NSD group and showed its three characteristic layers, namely, the outer polymorphic, middle pyramidal, and inner molecular layers. Most pyramidal cells were large and contained large vesicular nuclei. A few pyramidal cells were shrunken, exhibiting dark and condensed nuclei and deeply stained cytoplasm. Neuroglial cells and blood vessels were present in the neuropil (Figures 6c, 7c).

Regarding the SDIF group, it showed the normal histoarchitecture of this area of the hippocampus, including the three characteristic layers, namely, the polymorphic, pyramidal, and molecular layers. Some pyramidal cells appeared normal, whereas others were shrunken with darkly stained nuclei (Figures 6d, 7d).

The NSD group showed the fewest degenerated pyramidal cells, reflecting normal neuronal integrity in the CA1 region. The SD group showed a marked increase in degenerated pyramidal cells, with significant differences compared to that in NSD (*p* ≤ 0.001). Both the SDP and SDIF groups showed improvement relative to SD. The SDP group showed a substantial decrease in degenerated pyramidal cells, while the SDIF group also showed a decrease (*p* ≤ 0.001). Direct comparison revealed that SDP was significantly more effective than SDIF in reducing neuronal degeneration (*p* ≤ 0.001) (Figure 7e).

### 4.10 Cresyl violet-stained sections

**FIGURE 8:**
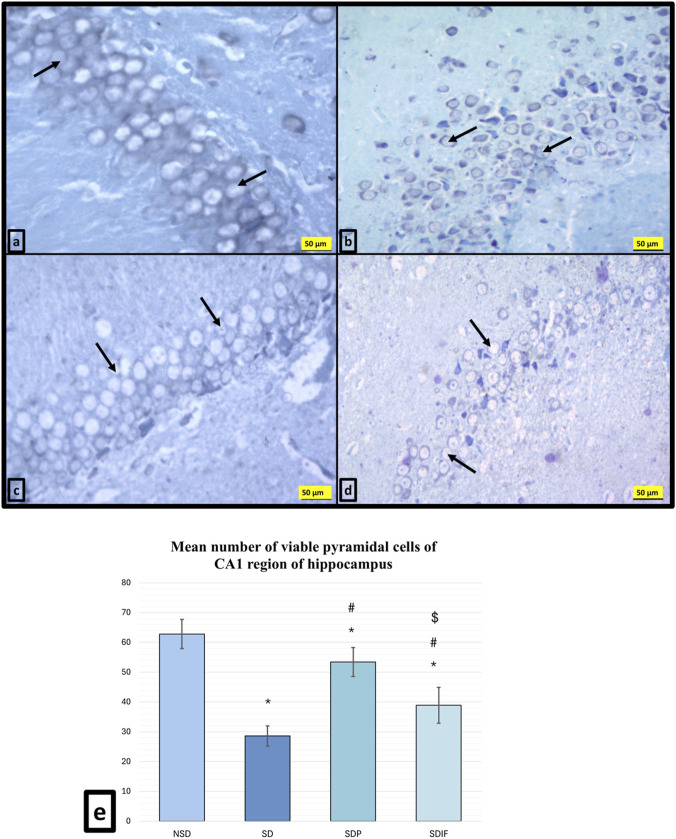
Cresyl violet-stained sections of the CA1 region of the hippocampus: **(a)** NSD group shows many viable pyramidal cells (arrows). **(b)** SD group shows a marked reduction in viable pyramidal cells (arrows). **(c)** SDP group shows a noticeable restoration in the number of cells relative to the SD group (arrows). **(d)** SDIF group showing a partial improvement, with the number of viable pyramidal cells increased relative to the SD group but lower than that observed in the SDP group (arrows). (Cresyl violet × 200, scale bar = 50 μm). **(e)** Mean number of viable pyramidal cells of the CA1 region of the hippocampus. *Significant versus the NSD group, #significant versus the SD group, and $ significant versus the SDP group, n = 10.

The NSD and SDP groups showed viable pyramidal neurons (Figures 8a, c). (Figure 8) The number of viable pyramidal neurons was lower in the SD group (Figure 8b). The number of viable pyramidal neurons increased in the SDIF group (Figure 8d). Compared to the NSD, morphometric examination of the mean number of viable pyramidal neurons showed a significant decrease (*p* ≤ 0.001) in the SD group, but the SDP group showed a substantial increment (*p* ≤ 0.001). The SDIF group showed an increase in viable cells relative to the SD group (p ≤ 0.001) and differed significantly from both the SDP and NSD groups (Figure 8e).

### 4.11 Immunohistochemistry findings

#### 4.11.1 Immunohistochemistry of GFAP

**FIGURE 9:**
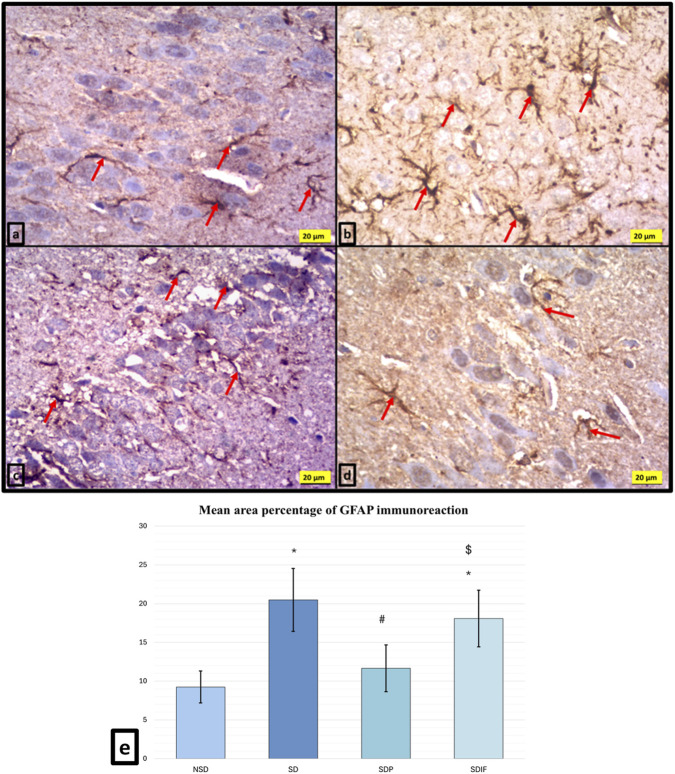
GFAP-immunostained sections of the CA1 region of the hippocampus: **(a)** NSD group shows protoplasmic astrocytes within the three hippocampal layers (red arrows). **(b)** SD group demonstrates marked upregulation of GFAP-positive protoplasmic astrocytes in all three layers (red arrows). **(c)** SDP group exhibits a noticeable downregulation of GFAP-positive astrocytes relative to the SD group (red arrows). **(d)** SDIF group shows a partial reduction in GFAP immunoreactivity, lower than that of the SD group but less reduced than that in the SDP group (red arrows). (GFAP immunostaining × 400, scale bar = 20 μm). **(e)** Mean area percentage of GFAP immunostaining in the CA1 region of the hippocampus. *Significant versus the NSD group, #significant versus the SD group, and $ significant versus the SDP group, n = 10.

GFAP-immunostained CA1 region of the sections of hippocampus of NSD and SDP groups revealed resting protoplasmic astrocytes in three layers of hippocampus (Figures 9a, c). (Figure 9) The SD group showed upregulation of the GFAP-positive reaction in the protoplasmic astrocytes of the hippocampus layers (Figure 9b). The SDIF group showed downregulation of the GFAP-positive reaction in the three layers of the hippocampus (Figure 9d).

Morphometric analysis of the mean area percentage of GFAP immunoreactivity revealed that GFAP expression increased in the SD group compared with that in the NSD group (*p* ≤ 0.001), indicating marked astrocytic activation. The SDP group showed a significant reduction in GFAP compared with that in the SD group (*p* ≤ 0.001), demonstrating a strong protective effect. The SDIF group also exhibited significantly lower GFAP expression than the SD group (*p* ≤ 0.001). A direct comparison between the SDP and SDIF groups revealed a significant difference (*p* ≤ 0.001), indicating that SDP was more effective than SDIF in reducing GFAP expression (Figure 9E).

#### 4.11.2 Immunohistochemistry of NF-κβ

**FIGURE 10:**
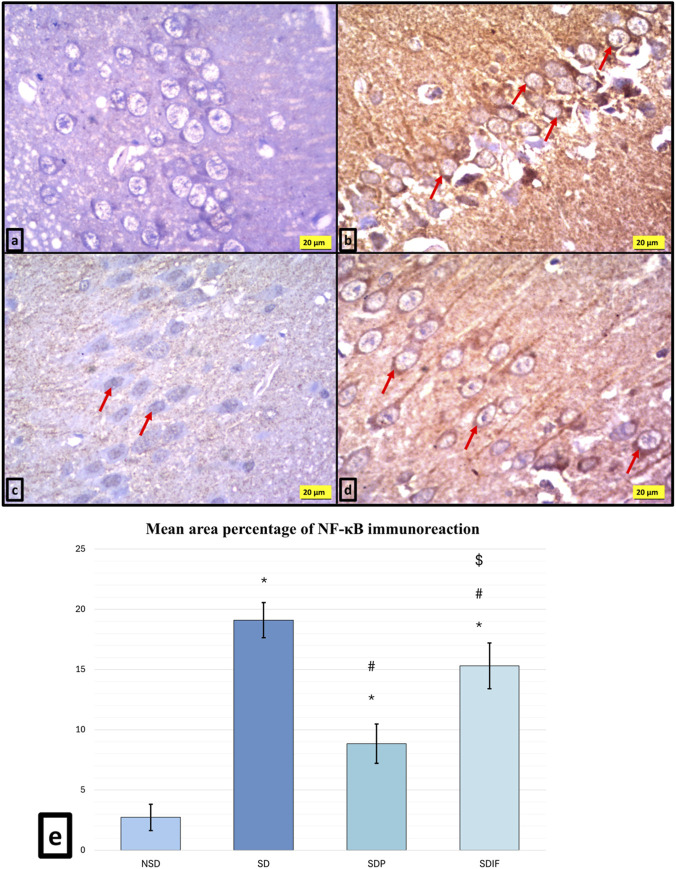
NF-κB-immunostained sections of the CA1 region of the hippocampus: **(a)** NSD group shows a negative cytoplasmic or nuclear expression in hippocampal pyramidal neurons. **(b)** SD group shows pyramidal neurons with strong cytoplasmic and nuclear immunoexpression (red arrows). **(c)** SDP shows faint nuclear expression in hippocampal pyramidal neurons (red arrows). **(d)** SDIF group shows some pyramidal neurons with moderate cytoplasmic and/or nuclear expression compared to the SD group (red arrows). (NF-κB immunostaining × 400, scale bar = 20 μm). **(e)** Mean area percentage of NF-κB immunostaining in the CA1 region of the hippocampus. *Significant versus the NSD group, #significant versus the SD group, and $ significant versus the SDP group, n = 10.

NF-κβ-immunostained CA1 region of the hippocampus sections of the NSD group revealed negative cytoplasmic and nuclear immunoreactivity in pyramidal neurons (Figure 10a). (Figure 10) In contrast, the SD group revealed marked NF-κB immunoexpression, with numerous pyramidal neurons exhibiting strong cytoplasmic and nuclear staining (Figure 10b). The SDP group showed mild nuclear immunoreactivity in pyramidal neurons (Figure 10c). In the SDIF group, NF-κB expression was moderate, with some pyramidal neurons displaying cytoplasmic and/or nuclear staining when compared to that in the SD group (Figure 10d).

Morphometric analysis of the mean area percentage of NF-κB immunoreactivity showed an increase in NF-κB expression in the SD group relative to the NSD group (*p* ≤ 0.001). The SDP group showed a decrease in NF-κB expression relative to the SD group (*p* ≤ 0.001). The SDIF group also revealed a decrease in NF-κB expression relative to the SD group (*p* ≤ 0.001); however, its expression remained significantly higher than that observed in the SDP group (*p* ≤ 0.001) (Figure 10e).

#### 4.11.3 Immunohistochemistry of TNF-α

**FIGURE 11:**
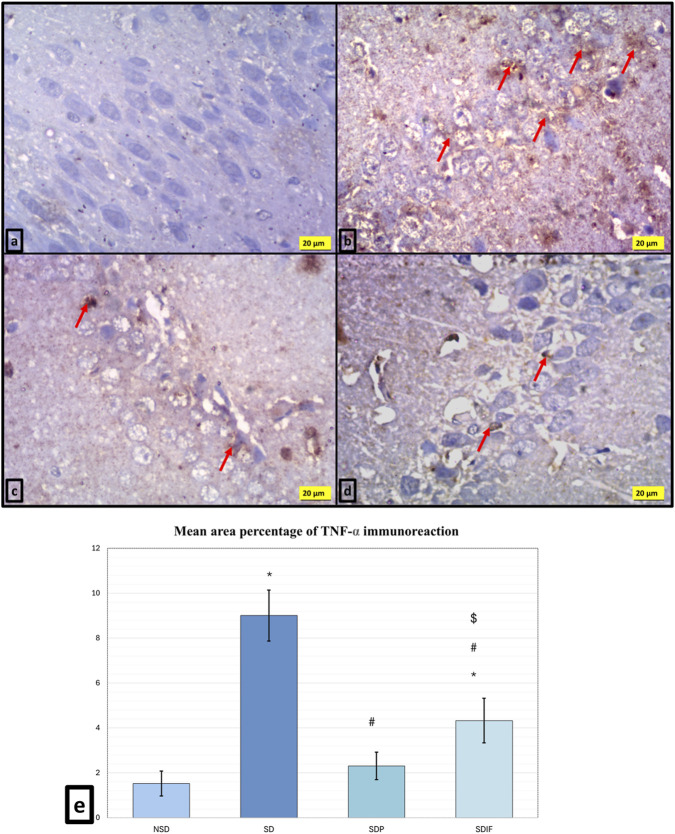
TNF-α-immunostained sections of the CA1 region of the hippocampus: **(a)** NSD group shows negative cytoplasmic expression in hippocampal pyramidal neurons. **(b)** SD group exhibits many pyramidal neurons with positive cytoplasmic expression (red arrows). **(c)** SDP group shows minimal cytoplasmic expression in hippocampal pyramidal neurons (red arrows). **(d)** SDIF group shows mild cytoplasmic immunoreactivity in pyramidal neurons (red arrows). (TNF-α immunostaining × 400, scale bar = 20 μm). **(e)** Percentage of TNF-α immunostaining in the CA1 region of the hippocampus. *Significant versus the NSD group, #significant versus the SD group, and $ significant versus the SDP group, n = 10.

TNF-α immunostaining of the CA1 region of the hippocampus in the NSD group showed negative cytoplasmic expression in hippocampal pyramidal neurons (Figure 11a). (Figure 11) In contrast, the SD group showed many pyramidal neurons with positive cytoplasmic expression (Figure 11b). The SDP group showed minimal cytoplasmic expression in pyramidal neurons (Figure 11c). According to the SDIF group, only a few pyramidal neurons exhibited faint cytoplasmic expression relative to the SD group (Figure 11d).

**FIGURE 12:**
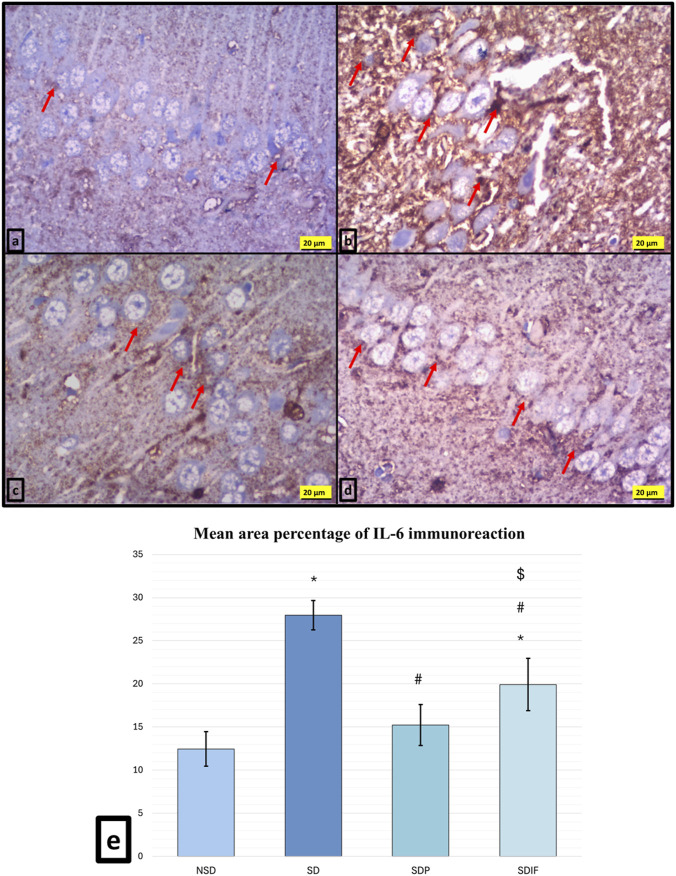
IL-6-immunostained sections of the CA1 region of the hippocampus: **(a)** NSD group shows minimal cytoplasmic expression in hippocampal pyramidal neurons (red arrows). **(b)** SD group exhibits many pyramidal neurons with strong positive cytoplasmic expression (red arrows). **(c)** NSP group shows mild cytoplasmic expression in hippocampal pyramidal neurons (red arrows). **(d)** SDIF group shows moderate cytoplasmic immunoreactivity in pyramidal neurons (red arrows). (IL-6 immunostaining × 400, scale bar = 20 μm). **(e)** Mean area percentage of IL-6 immunostaining in the hippocampus. *Significant versus the NSD group, # significant versus the SD group, and $ significant versus the SDP group, n = 10.

Morphometric analysis of the mean area percentage of TNF-α immunoreactivity showed that TNF-α expression increased in the SD group relative to the NSD group (*p* ≤ 0.001). The SDP group showed a decrease in TNF-α expression relative to the SD group (*p* ≤ 0.001). The SDIF group also showed a decrease in TNF-α expression relative to the SD group (*p* ≤ 0.001); however, its expression is significantly higher than that observed in the SDP group (*p* ≤ 0.001). Direct comparisons among all groups revealed statistically significant differences, indicating that the SDP group exerted a stronger anti-inflammatory effect than the SDIF group (Figure 12e).

#### 4.11.4 Immunohistochemistry of IL-6

IL-6 immunostaining in the NSD group revealed minimal cytoplasmic expression in the hippocampal pyramidal neurons (Figure 12a). (Figure 12) In contrast, the SD group exhibited several pyramidal neurons with positive cytoplasmic expression (Figure 12b). The SDP group showed mild cytoplasmic expression in hippocampal pyramidal neurons (Figure 12c). Regarding the SDIF group, moderate cytoplasmic immunoreactivity was observed in hippocampal pyramidal neurons (Figure 12d).

Morphometric analysis of the mean area percentage of IL-6 immunoreactivity revealed that IL-6 expression was increased in the SD group relative to the NSD group (*p* ≤ 0.001). The SDP group showed a reduction in IL-6 expression relative to the SD group (*p* ≤ 0.001). The SDIF group also showed a significant decrease in IL-6 expression relative to the SD group (*p* ≤ 0.001); however, its expression remained higher than that observed in the SDP group (*p* ≤ 0.001) (Figure 12e).

#### 4.11.5 Immunohistochemistry of cleaved caspase-3

**FIGURE 13:**
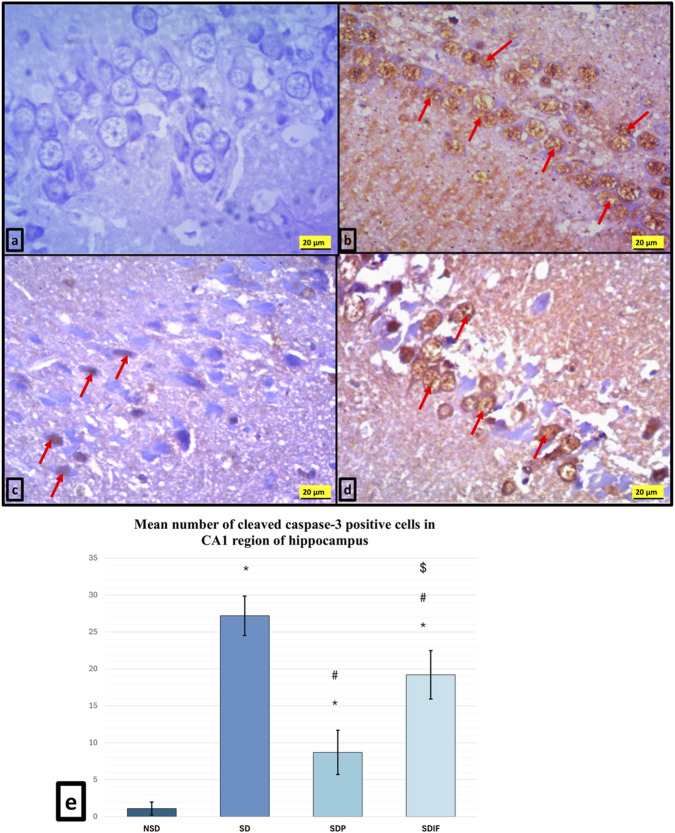
Cleaved caspase-3-immunostained sections of the CA1 region of the hippocampus: **(a)** NSD group shows a negative nuclear reaction for cleaved caspase-3. **(b)** SD group shows numerous cleaved caspase-3-positive pyramidal neurons’ nuclei (red arrows). **(c)** SDP group shows a few pyramidal neurons with cleaved caspase-3-positive nuclei (red arrows). **(d)** SDIF group exhibits many cleaved caspase-3-positive pyramidal neurons’ nuclei (red arrows). (Cleaved caspase-3 immunostaining×400, scale bar = 20 μm). **(e)** Mean number of cleaved caspase-3-positive cells in the CA1 region of hippocampus. * Significant versus the NSD group, # significant versus the SD group, and $ significant versus the SDP group, n = 10.

Cleaved caspase-3-immunostained sections of the CA1 region of the hippocampus from the NSD group demonstrated a negative nuclear reaction for cleaved caspase-3 (Figure 13a). (Figure 13) In contrast, the SD group exhibited numerous cleaved caspase-3- positive nuclei (Figure 13b). Few cells with cleaved caspase-3- positive nuclei were observed in the SDP group (Figure 13c). In the SDIF group, many cells showed cleaved caspase-3-positive nuclei (Figure 13d).

Morphometric analysis of the mean area percentage of cleaved caspase-3 immunoreactivity revealed that cleaved caspase-3 expression was increased in the SD group relative to the NSD group (*p* ≤ 0.001), indicating enhanced apoptotic activity. While the SDP group showed a significant reduction relative to the SD group (*p* ≤ 0.001). The SDIF group also showed a significant decrease relative to the SD group (*p* ≤ 0.001); however, it has remained higher than that observed in the SDP group (*p* ≤ 0.001). Overall, the SDP group demonstrated a more pronounced anti-apoptotic effect than SDIF (Figure 13e).

## 5 Discussion

The findings of this study showed that SD led to abnormal neuropsychological behavior, impaired working spatial memory, and lower muscular endurance. SD impaired endurance performance, which was evidenced by reduced swimming time, and it is consistent with the studies of Nollet et al. (2020) and Chung et al. (2023). Higher serum levels of MDA, IL-6, and TNF-α and lower hippocampal SOD activity were associated with the effects that cause inflammation and oxidative stress. SD makes it difficult to adjust to new environmental challenges and stressors, which can lower the athletic performance (Charest and Grandner, 2020). Probiotic supplementation significantly improved the deterioration in endurance caused by SD, which could be explained by the enhanced antioxidant activity and GBA regulation. These findings were in agreement with the results of Wu et al. (2025), who found that probiotics may potentiate physical activity by modulating gut microbiota-derived metabolites, particularly SCFAs, which play an important role in GBA signaling, energy metabolism, and enhancing antioxidant capacity. The neuroprotective and systemic benefits observed in the SDP group underscore the importance of the specific probiotic strains selected for this study. The choice of *L. fermentum* and *L. delbrueckii* was not arbitrary; rather, it targeted the dual pathways of the microbiome–GBA disrupted by chronic SD. Our findings align with those of Liu et al. (2019), which indicate that *L. fermentum* likely mitigated cognitive deficits by suppressing pro-inflammatory cytokines (e.g., TNF-α and IL-6) and reducing oxidative stress in the hippocampus. Simultaneously, the inclusion of *L. delbrueckii* addressed the ‘leaky gut’ phenomenon typically induced by SD by reinforcing tight junction proteins and maintaining intestinal barrier integrity, as evidenced by Azagra-Boronat et al. (2020). This strain likely prevented the systemic translocation of lipopolysaccharides (LPS). Consequently, this synergistic combination provided a robust defense against both the central neuroinflammation and the peripheral metabolic disturbances that characterize chronic sleep loss. The strain has previously been demonstrated to have psychotropic properties by altering the serotonergic pathway during stress conditions in rats (Lin J. et al., 2023). While IF has the potential to enhance endurance and metabolic efficiency under baseline conditions (Peos et al., 2021), SD significantly compromise the neural drive and perception of effort (Rault et al., 2020), increases the basal metabolic rate and total energy expenditure (Menezes et al., 2020), and disturbs circadian regulation of glucose and lipid metabolism (Liu et al., 2024). These factors counteract the established metabolic and performance benefits of IF, leading to the lack of increase of SDIF rats’ endurance. SD-induced abnormal neuropsychological behavior appeared in the decreasing total entries to the center, decreasing time spent in the inner zone, decreasing total distance moved in the open field, increasing rearing frequency, increasing grooming episodes, and increasing freezing episodes. In addition, SD changed how the rats behaved in the Y-maze, which depends on the rats’ tendency to naturally explore novel environments rather than return to the one just visited. SD impaired the spatial working memory, as shown by decreased spontaneous alternation. In addition, SD induced behavioral despair, which was represented by increased immobility time in TST. These findings could be explained by the oxidative stress response. Oxidative stress could significantly affect CNS functions through mitochondrial dysfunction, interference with neuronal transmission, synaptic impairment, and suppression of neurogenesis (Ansari et al., 2023). The brain’s poor enzymatic antioxidant defense mechanisms and abundance of polyunsaturated fatty acids, unsaturated bonds in lipids, and greater consumption of oxygen make the brain more vulnerable to oxidative stress than other tissues (Lee K. H. et al., 2020; Feng et al., 2023). Neuroinflammation can explain the effect of SD on neuropsychological impairment. The increase in inflammatory marker levels such as IL-6 and TNF-α in the serum was monitored by pronounced Toll-like receptor 4 (TLR4) expression following both acute and chronic SD (Carroll et al., 2015). These results agree with the findings of Shaikh et al. (2024) and Ballesio et al. (2025), who reported that individuals with poor sleep quality had increased levels of IL-6, which negatively affect cognitive performance. Decreased SCFAs levels reflect that SD induced gut microbiota dysbiosis, which was known to modulate neuroplasticity and impaired GBA (Sun et al., 2023; Wang et al., 2023b). GBA is “bidirectional communication between the central and enteric nervous system, linking cognitive and emotional centers of the brain with intestinal function” (Clayton et al., 2021). The body’s circadian system is organized into a hierarchical structure led by the suprachiasmatic nucleus (SCN) in the hypothalamus. As the autonomous “master clock,” the SCN synchronizes with external light via retinal cells and coordinates peripheral clocks across the body through neural pathways and hormonal cues, such as the HPA axis (Hastings et al., 2018).

At the cellular level, this rhythm is maintained by a molecular feedback loop involving transcription factors such as *CLOCK* and BMAL1. These proteins initiate the production of *CLOCK* genes that eventually suppress their own synthesis, creating a self-sustaining 24-h cycle that is present in nearly all tissues (Kim et al., 2021). To our knowledge, research assessing changes in circadian rhythms caused by intermittent fasting are scarce. The study primarily focuses on general CNS–microbiota correlations but does not account for the specific regulatory functions of the hypothalamus. Furthermore, because intestinal tissues were not evaluated, the mechanisms underlying the observed bidirectional communication remain partially unaddressed, forming a basis for future studies (Lee J. H. et al., 2020).

SD dysregulated the *CLOCK* circadian gene in the hippocampus. The findings agreed with the results of Ke et al. (2022), who found that SD significantly changed seven circadian genes, which were strongly correlated with impaired spatial memory performance. Probiotics significantly reduced depressive-like immobility while improving neurobehavioral parameters, including locomotor activity and spontaneous alteration. The results agreed with the findings of Griffin et al. (2023). Probiotics’ anti-inflammatory and antioxidant properties can explain these results, so probiotics can improve memory consolidation, maintain hippocampus neuronal integrity, and decrease microglial activation, all of which help to maintain neuroimmune balance (Zheng et al., 2023). Probiotics treatment significantly decreases pro-inflammatory cytokines and nitric oxide and significantly improves spatial memory performance in lipopolysaccharide (LPS)-induced mice according to the findings of Lee et al. (2023). Restoring SCFAs levels with probiotics administration indicates better metabolic activity and gut microbiota diversity, which could play a crucial role in decreasing microglial activation and improving neural energy metabolism (Cao et al., 2025). The restoration of circadian rhythms is shown by the normalization of hippocampus *CLOCK* gene expression, which may be enhanced by metabolites produced from the microbiota, such as SCFAs and tryptophan–serotonin pathways (Cheng et al., 2025). SCFAs such as butyrate are known histone deacetylase inhibitors that can epigenetically control the expression of the *CLOCK* gene, thus realigning circadian transcriptional cycles and reestablishing hypothalamic function (Zhang et al., 2025). While IF was not as effective as probiotics, IF presents improvements in neurobehavioral parameters. These findings agree with the findings of Zhao Y. et al. (2025), who reported that in Alzheimer’s patients, TRF was associated with improved executive function and reduced cognitive decline, indicating the potential role of fasting in managing SD-related neurobehavioral impairments. These findings were supported by research demonstrating that IF can enhance synaptic plasticity and neurogenesis using BDNF/CREB signaling while also reducing pro-inflammatory cytokines and oxidative stress (Mayor, 2023). IF can activate autophagy, thereby removing damaged organelles and protein aggregates that accumulate following chronic sleep restriction (Shabkhizan et al., 2023). Fasting also helps create a metabolic shift toward ketone utilization, which provides an efficient energy source for the brain and enhances neuronal resilience. The GBA also contributes to IF remodeling ability of gut microbiota composition, restores diurnal microbiota oscillations, and increases production of SCFAs, which helps to regulate *CLOCK* gene expression and suppress microglial activation (Zeb et al., 2023; Loh et al., 2024). These findings agreed with the results of Whittaker et al. (2023) and Helbling et al. (2024), who concluded that in mouse models of neurodegeneration associated with sleep loss, TRF restored rhythmic gene expression in the hippocampus, reduced neuroinflammation, and improved spatial memory and recognition. SD causes major structural, inflammatory, and apoptotic changes in the hippocampal CA1 region, while probiotics and IF ameliorate these effects to varying degrees according to histological and immunohistochemical analyses. Despite the valuable insights gained from this study, it is important to acknowledge the inherent limitations of using an animal model. While rats provide a controlled environment to study the effects of probiotics or IF on SD, they do not fully resemble the complex physiological and genetic landscape of human biology, so further research is required for the findings to be clinically applied.

Histological and immunohistochemical analyses of the hippocampal CA1 region demonstrate that SD induced significant structural, inflammatory, and apoptotic changes, whereas probiotics and IF mitigated these effects to varying extents.

The NSD condition maintained normal CA1 cytoarchitecture with clearly defined polymorphic, pyramidal, and molecular layers and densely packed pyramidal neurons, reflecting preserved neuronal integrity. In contrast, SD induced histological changes in the form of a loss of hippocampal tissue integrity. The CA1 region showed that the pyramidal cells are degenerated with shrunken hyperchromatic nuclei and perinuclear halos. These findings agree with Konakanchi et al. (2023), who found that 21 days of chronic SD-induced changes in the hippocampus might be the reason for the rats’ impairment of the spatial memory performance. Morphometric analysis of the mean number of degenerated and viable pyramidal cells revealed that SD led to an increase in degenerated cells and a decrease in healthy neurons compared to that in the NSD group. When probiotics or IF were presented with SD, neuronal viability improved, with the probiotic group showing the strongest protective effect, which is consistent with literature indicating that metabolic and microbiota-modulating interventions can enhance hippocampal resilience via neurotrophic and anti-inflammatory pathways (Ebrahimi et al., 2025; Hein et al., 2025). Immunohistochemical findings revealed an increase in GFAP levels in the SD group, indicating increased astrocyte activity and gliosis, which are common reactions to stress in the central nervous system and markers of ongoing neuroinflammation. Both probiotic and IF treatments led to a decrease in GFAP expression, indicating the attenuation of astrocytic activation (Won et al., 2025; Zhao Z. et al., 2025). SD led to an increase in NF-κB, a key regulator in driving inflammation, within the pyramidal neurons. Probiotics and IF helped suppress NF-κB, with probiotics having the strongest effect. This indicates that these strategies can effectively modulate the inflammatory signaling pathways (Lee et al., 2024). SD increased the surge in pro-inflammatory cytokines, specifically TNF-α and IL-6, in the hippocampal CA1 area. This indicated a strong activation of the brain’s immune defenses and inflammation. When probiotics or IF were added to the SD groups, the levels of TNF-α and IL-6 decreased significantly compared to that with SD alone, though they did not return to the levels seen in the NSD group. These results indicate that probiotics and IF modulate rather than prohibit the inflammation in the hippocampus, maintaining the balance of the immune system. This agrees with recent research showing that the GBA is responsible for immune activity in the brain, cutting down on harmful inflammation and protecting nerve cells (Nakhal et al., 2024; Rathor and Ch, 2024). Cleaved caspase-3, a well-known marker of cell apoptosis, was found at much higher levels in hippocampal CA1 neurons after SD, highlighting an increase in programmed cell death. Probiotics or IF help in decreasing caspase-3 levels, along with with the strongest protective effect against cell loss. These results support previous research indicating that dietary changes and probiotics can protect neurons by influencing cell death pathways and reducing oxidative stress and inflammation in the brain (Xiao et al., 2025). Probiotic supplementation maintained hippocampal integrity, with intact pyramidal layers and reduced vacuolations, indicating neuroprotection. Probiotics attenuated brain pro-inflammatory cytokine expression and oxidative damage, restored hippocampus-dependent behavioral performance, and normalized molecular markers of synaptic plasticity (e.g., BDNF) (Zheng et al., 2023; Tian et al., 2024). These biochemical and functional improvements can lead to the preservation of hippocampal cellular morphology and neuropil integrity (Salami and Soheili, 2022). In addition, probiotics reduced astrocyte activation and microglial reactivity and decreased apoptosis, as indicated by reduced GFAP and caspase-3 expression. These findings agree with Faccinetto-Beltrán et al. (2024), who found that in a cognitive impairment rat model, combined treatment including probiotics significantly reduced GFAP-positive astrocyte activation in the hippocampal regions, and Karakayalı et al. (2023), who documented the anti-apoptotic role of probiotics in brain injury models. IF produced partial histological improvement, but mild degenerative changes persisted. These results agree with Lee et al. (2024), who found that IF can enhance adult hippocampal neurogenesis, increase markers of synaptic plasticity (e.g., BDNF and synaptophysin), upregulate stress-resilience pathways (e.g., SIRT3 and Klotho), preserve neuronal morphology, and improve neuropil organization. IF reduces hippocampal neuroinflammation through lowering pro-inflammatory cytokines and decreases oxidative damage in hippocampal tissue in models of high-fat diet and infection (Vasconcelos et al., 2014; Lee et al., 2024).

Previous research indicates that IF may enhance sleep quality through two primary physiological mechanisms, namely, A) circadian optimization: restricting food intake during nocturnal hours strengthens peripheral circadian rhythms. This alignment helps restore the internal homeostatic balance, which is particularly beneficial for individuals with irregular sleep–wake cycles. B) Weight management: by facilitating weight loss, IF improves core sleep metrics, including duration and efficiency, and mitigates the risk factors associated with obstructive sleep apnea (McStay et al., 2021). Moreover, IF can improve cognitive and learning abilities in rat models with the activation of antioxidant enzymes and autophagy to resist cellular stress (Longo and Panda, 2016).

## 6 Conclusion

In the present study, SD significantly impaired exercise performance, neurobehavioral outcomes, circadian rhythmicity, gut microbiota balance, and hippocampal structure. Both probiotics and IF ameliorated these impairments, but probiotics were superior in most parameters through modulating the *CLOCK* gene as a provisional study via many anti-inflammatory and antioxidant mechanisms, underscoring the therapeutic value of targeting the GBA in chronic SD. Therefore, probiotics and IF could be recommended to control stress-related disorders. Histological examination further revealed that chronic SD induced marked structural alterations in the hippocampus, including neuronal degeneration and disrupted cellular organization. Notably, both probiotics and IF mitigated these histopathological changes, with probiotics demonstrating more pronounced neuroprotective effects. These findings highlight the potential of probiotics and IF in preserving the brain architecture under conditions of stress. Therefore, probiotics and IF could be recommended as promising strategies to control stress-related disorders.

## 7 Limitations of the study

A key limitation of the present work is that *CLOCK* gene expression was measured at a single time-point and without parallel assessment of other core circadian regulators (e.g., BMAL1, PER, and CRY). Therefore, the results do not allow evaluation of gene rhythmicity, phase shifts, or 24-h oscillatory patterns. Although the *CLOCK* findings provide preliminary insight into SD-related hippocampal molecular changes, they should be interpreted cautiously. Future studies should incorporate multiple sampling points across the circadian cycle and a panel of clock genes to fully characterize SD-induced circadian disruption. Another limitation of this study is that it was carried out as a single experimental repetition. It is common in controlled animal experiments but limits the assessment of inter-experimental variability. While the sample size per group was adequate for the statistical analyses, the absence of independent experimental replicates means that the reported standard deviations do not account for repetition variability. Future studies with multiple independent cohorts will be valuable for confirming the robustness and reproducibility of the observed effects.

## Data Availability

All data generated or analysed during this study are included in this published article and its supplementary information files.
